# Exome sequencing of choreoacanthocytosis reveals novel mutations in *VPS13A* and co-mutation in modifier gene(s)

**DOI:** 10.1007/s00438-023-02032-2

**Published:** 2023-05-20

**Authors:** Sima Chaudhari, Akshay Pramod Ware, Dushyanth Babu Jasti, Sankar Prasad Gorthi, Lavanya Prakash Acharya, Manoj Bhat, Sandeep Mallya, Kapaettu Satyamoorthy

**Affiliations:** 1 Department of Cell and Molecular Biology, Manipal School of Life Sciences, Manipal Academy of Higher Education, Manipal, Karnataka 576104 India; 2 Department of Bioinformatics, Manipal School of Life Sciences, Manipal Academy of Higher Education, Manipal, Karnataka 576104 India; 3 Department of Neurology, Kasturba Medical College, Manipal, Karnataka 576104 India; 4 Department of Neurology, Bharati Hospital and Research Center, Bharati Vidyapeeth (Deemed to be University) Medical College and Hospital, Dhankawadi, Pune, Maharashtra 411043 India

**Keywords:** Choreoacanthocytosis, *VPS13A*, Whole exome sequencing, Mitochondria localization, Mutation, TOMM40

## Abstract

Choreoacanthocytosis, one of the forms of neuroacanthocytosis, is caused by mutations in vacuolar protein sorting-associated protein A (*VPS13A*), and is often misdiagnosed with other form of neuroacanthocytosis with discrete genetic defects. The phenotypic variations among the patients with *VPS13A* mutations significantly obfuscates the understanding of the disease and treatment strategies. In this study, two unrelated cases were identified, exhibiting the core phenotype of neuroacanthocytosis but with considerable clinical heterogeneity. Case 1 presented with an additional Parkinsonism phenotype, whereas seizures were evident in case 2. To decipher the genetic basis, whole exome sequencing followed by validation with Sanger sequencing was performed. A known homozygous pathogenic nonsense mutation (c.799C > T; p.R267X) in exon 11 of the *VPS13A* gene was identified in case 1 that resulted in a truncated protein. A novel missense mutation (c.9263T > G; p.M3088R) in exon 69 of *VPS13A* identified in case 2 was predicted as pathogenic. In silico analysis of the p.M3088R mutation at the C-terminus of VPS13A suggests a loss of interaction with TOMM40 and may disrupt mitochondrial localization. We also observed an increase in mitochondrial DNA copy numbers in case 2. Mutation analysis revealed benign heterozygous variants in interacting partners of VPS13A such as *VAPA* in case 1. Our study confirmed the cases as ChAc and identified the novel homozygous variant of *VPS13A* (c.9263T > G; p.M3088R) within the mutation spectrum of *VPS13A-*associated ChAc. Furthermore, mutations in *VPS13A* and co-mutations in its potential interacting partner(s) might contribute to the diverse clinical manifestations of ChAc, which requires further study.

**Supplementary Information:**

The online version contains supplementary material available at 10.1007/s00438-023-02032-2.

## Introduction

Choreoacanthocytosis (ChAc) or VPS13A disease is the major form of a rare group of neurodegenerative disorder called “Neuroacanthocytosis” and has been recently categorized as a form of proteinopathies and VPS13opathies (Velayos Baeza et al. 2019; Walker and Danek 2021; Peikert et al. 2022). Presence of thorny red blood cells in the peripheral blood smear, neurodegeneration of the basal ganglia, uncontrollable hyperkinetic movements, cognitive impairment, and neuropsychiatric features are the core features of ChAc. However, additional phenotypes such as epilepsy, self-mutilation behavior, bruxism, dysarthria, dysphasia, seizure attacks, parkinsonian symptoms, and diminished or reduced tendon are also observed (Velayos Baeza et al. 2019; Shen et al. 2017; Mitchell et al. 2021). Magnetic resonance imaging (MRI) shows atrophy of the striatum-caudate nuclei, putamen, globus pallidum, and substantia nigra and, occasionally, deposition of iron (Velayos Baeza et al. 2019; Shen et al. 2017; Niemelä et al. 2020). ChAc is a genetic disorder with an autosomal recessive inheritance pattern and a progressive disease course (Danek et al. 2012). Mutations in the vacuolar protein sorting-associated protein A (*VPS13A*) are associated with ChAc (Ueno et al. 2001; Rampoldi et al. 2001); however, the clinical phenotype differs among the cases even with the same mutation, suggesting complex gene–gene interactions or gene–environment interactions as modifying factors (Niemelä et al. 2020; Tomiyasu et al. 2012). The spectrum of mutations in *VPS13A* gene explains some but not all of the described phenotypes of ChAc, and this heterogeneity remains to be fully explored.

*VPS13A* is a multi-exonic gene spanning 240 kb consisting of 73 exons with four major alternatively spliced transcripts (Rampoldi et al. 2001; Dobson-Stone et al. 2002; Velayos-Baeza et al. 2004). Various mutations, inclusive of missense, nonsense, frameshift, duplication, deletion, and splice site mutations, have been reported throughout the *VPS13A* gene in ChAc patients (Shen et al. 2017; Dobson-Stone et al. 2002). Hence, variants are not confined to a particular domain but are distributed throughout the VPS13A protein. The ClinVar database reports 329 variants alone that affect the protein coding regions (www.ncbi.nlm.nih.gov/clinvar/). Reduced or absence of VPS13A protein (chorein) resulting in loss of function underpins this recessive disorder. Several reported pathogenic missense mutations affect the residues that are relevant for the stability and function of VPS13A (Masana et al. 2021). *VPS13A* being a large gene, whole exome sequencing (WES) is advantageous for the identification of unknown mutations (Gilissen et al. 2012; Fridman et al. 2021). Here, we studied two unrelated subjects with ChAc disorder but different clinical phenotypes. WES followed by Sanger sequencing was employed to identify the underlying genetic causes of the phenotypes. Further, WES data were analyzed for the spectrum of co-mutations that may be responsible for the diverse phenotype among the ChAc patients with the *VPS13A* mutation.

## Methods

### Patient recruitment and ethical consideration

The cases were recruited during the study on neurodegenerative disorders approved by the Ethical Committee, Kasturba Hospital/Kasturba Medical College, Manipal (registry no. IEC365/2017; CTRI/2017/07/00904). Blood samples and clinical details were collected from the patient upon obtaining written informed consent.

### Whole exome sequencing (WES) and data analysis

DNA extraction and WES were performed as previously described by Chakrabarty et al. (2021) using the Ion Torrent platform (ThermoFisher Scientific, USA). Pre-processing and annotation of sequenced data were done with Torrent suite software (ThermoFisher Scientific, USA) and Ion Reporter software V5.18 (Thermo Fisher Scientific, USA), respectively. Cascade of filtration criteria such as minor allele frequency of < 1%, non-synonymous, homozygous and heterozygous mutations were included for the prioritization of variants. From each case’s data, the variants present in the pool of our in-house normal exome data were excluded. The remaining variants were further shortlisted when their coverage was > 10 × , present within the homopolymer region (< 4), absent in UCSC common SNP database and when the variants were expressed in brain. The UMD-Predictor tool associated with Variant Annotation and Filter Tool (VarAFT) software was employed for pathogenicity prediction (Desvignes et al. 2018). The homozygous and compound homozygous variants in genes previously associated with the neurodegenerative disorders were analyzed. Additionally, variants in the genes that encode the interacting partners of identified candidate genes or genes of the same protein class were also shortlisted.

### Sanger sequencing

The targeted regions were amplified using primers as described in Online Resource 1 and further sequenced using the big dye terminator kit in ABI 3130 Genetic analyzer (Applied Biosystems, Monza, Italy).

### Mitochondrial DNA (mtDNA) copy number analysis by qPCR

The relative copy number of mtDNA was estimated using the protocol described by Ashar et al. (2015) with slight modifications. FAM-labeled primer targeting to a mitochondrial gene NADH dehydrogenase 1 (*ND1*) (Hs02596873_s1), and a VIC labeled primer specific to a single copy Ribonuclease P RNA component H1 region (*RPPH1*) of the nuclear genome (TaqMan copy number reference assay, human, RNase; 4403326) were used in the real-time quantitative polymerase chain reaction (qPCR) and TaqMan chemistry (Applied Biosystems). ΔCt value and 2^−ΔCt^ were calculated, and the standard deviation of ΔCt values of the two replicates was estimated.

### Computational analysis

Ortholog of human VPS13A in representative or model organisms from each phylum of eukaryotes was obtained from UniProt (www.uniprot.org/) and the NCBI protein database (www.ncbi.nlm.nih.gov/protein/) for multiple sequence alignment using the Clustal Omega tool (Sievers and Higgins 2018). The homology of VPS13A region containing identified ChAc mutations was used to obtain Logo diagram using the WebLogo 3 program (Crooks et al. 2004). The interacting proteins for VPS13A were shortlisted from the Human Integrated Protein Protein Interaction rEference (HIPPIE) database via the open-source framework Network Data Exchange (NDEx) network biological repository (Pratt et al. 2015). The expression data for various isoforms of VPS13A in different human organs were obtained from the Genotype-Tissue Expression (GTEx) database (www.gtexportal.org/home/gene/VPS13A). The proteins of the same family class were identified with the PANTHER database (www.pantherdb.org/). The predicted structures of human translocase of outer mitochondrial membrane (TOMM)20 and TOMM40 were obtained from the Alphafold database (Varadi et al. 2022).

### Hydrophobicity plot

Alteration in the hydrophobic regions in protein was detected with Kyte–Doolittle scale in Protscale (www.web.expasy.org/protscale/).

### Protein modeling

The ab initio 3D protein structure for the C-terminus of hVPS13A (2615–3174aa) was predicted using the trRosetta algorithm (Du et al. 2021). The mutant version of the C-terminus was modeled in Chimera 1.1 (Pettersen et al. 2004) using homology modeling approach where the ab initio structure generated from trRosetta was used as a template. The structures were further assessed using the structure assessment tool in the Swiss-Model interface (www.swissmodel.expasy.org/assess). Chimera 1.15 was used to perform superimposition and visualization for differences in protein conformation caused by mutation.

### Protein–protein docking

PatchDock was used for protein–protein docking (Schneidman-Duhovny et al. 2005). Clustering root mean square deviation (RMSD) of 4 and default complex types were selected. Refinement and rescoring of the protein–protein docking solution from PatchDock were performed using FireDock (Mashiach et al. 2008). Visualization and assessment of interaction were performed in Pymol (PyMOL Molecular Graphics System, Version 2.0 Schrödinger, LLC).

## Results

### Clinical assessment and mutation analysis

#### Case 1

**Fig. 1:**
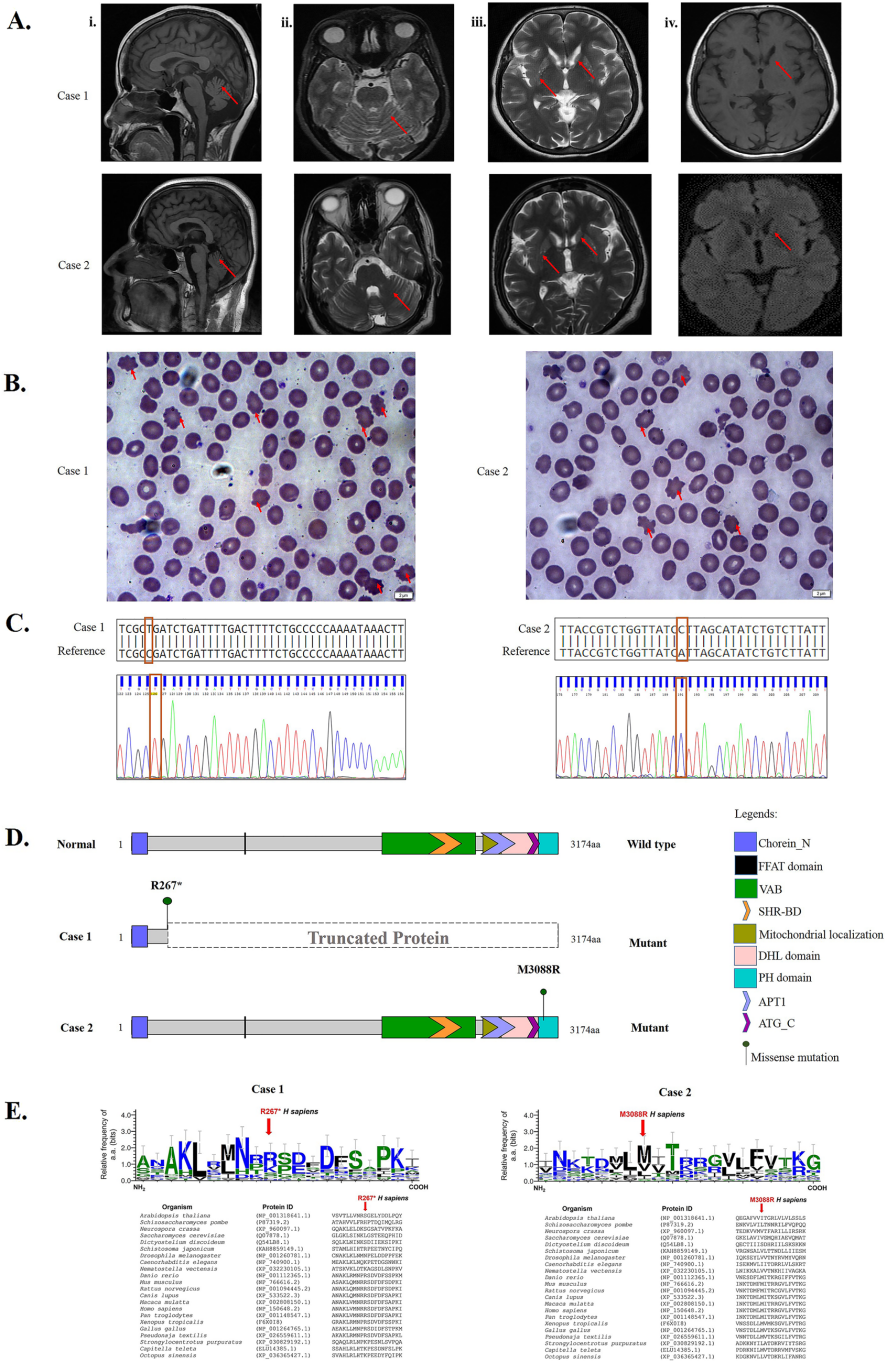
Clinical and molecular finding in two cases of choreoacanthocytosis: **A** Brain MRI of cases. Sagittal T1-weighted brain MRI (i) and axial FLAIR-weighted brain MRI (ii, iii, iv) indicated marked cerebellar atrophy, atrophy of the putamen and caudate nuclei. **B** Peripheral blood smear showing acanthocytes (marked by arrow) in cases. **C** Exome sequencing followed by confirmation by Sanger sequencing revealed homozygous c.799C > T; p.R267* in case 1 and c.9263T > G; p.M3088R in case 2. **D** The mutation in case 1 results in premature termination of the VPS13A protein, whereas the mutation at the conserved residue identified in case 2 is at the C-terminus region of the VPS13A responsible for mitochondrial localization. **E** Logo plot showing the sequence conservation of VPS13A residues (mutated in cases 1 and 2) among the eukaryotes

A 47-year-old lady visited the Department of Neurology, Kasturba Hospital, Manipal, with complaints of change in physical behavior over a period of 3 years, slowness of gait, and daily activities in the past 18 months. The patient had neither a premorbid illness nor family history of the illness. On examination, involuntary movements were noted in the right limb. Choreiform movements were observed in face and tongue. The patient also presented monotonous speech, hyperreflexia in bilateral upper and lower limbs, bradykinesia, and cogwheel rigidity. Parkinsonism symptoms were more prevalent on the right than on the left. Electromyography (EMG) showed mild bilateral lower limb axonal motor neuropathy. The Mini-Mental State Exam (MMSE) test showed the score of 27/30 and impaired frontal lobe functions, along with symptoms of apathy and depression. The laboratory inspection for ALT (alanine transaminase) and AST (aspartate amino transferase) using the IFCC method without pyridoxal phosphate (UV kinetics; Roche Diagnostics) showed normal levels of 9.0 and 27.0 IU/L, respectively. Creatinine phosphokinase (CPK) estimation by creatine kinase, activated by N-Acetyl Cysteine (UV kinetics; Roche Diagnostics) method, and lactate dehydrogenase estimation (UV assay; Roche Diagnostics) revealed normal levels of 56 U/L and 210 IU/L, respectively (Table 1). MRI of the brain showed symmetrical atrophy of bilateral putamen and caudate nuclei with mildly increased T2W and a FLAIR signal with subtle iron deposition (Fig. 1A). Peripheral blood smears followed by Leishman staining showed the presence of acanthocytes (Fig. 1B). WES revealed a stop-gain mutation (c.799C > T; p.R267X) in the *VPS13A* gene while no mutation was found in the *XK* gene, confirming the patient as a case of ChAc and overruling the possibility of McLeod syndrome.

**Table 1:** Diagnostic evidence observed in the presented ChAc cases

	Case 1	Case 2
Gender/age at onset	Female/44	Male/25
Family history	Absent	Absent
Primary symptoms	Behavioral disturbance, slowness of daily activities, slowness in gait	Seizure episodes
Incipient symptom	Involuntary movements of right upper limb and lower limb	Involuntary movements of jaw, tongue with swallowing difficulty
Dystonia distribution	Upper limbs, tongue	Tongue, upper limbs
Chorea distribution	Face, tongue	Face, perioral region, tongue
Dysarthria	+	+ +
Tongue and lip biting	+	+ +
Parkinsonian features	+ + +	Absent
Tendon reflex	Hyperreflexia bilateral upper limb and lower limb	Hyporeflexia bilateral upper limb and lower limb
Seizure type	Absent	Generalized tonic–clonic seizures
Cognitive impairment	Frontal lobe dysfunction (+ +)	Frontal lobe dysfunction (+ +)
Psychiatric symptoms	Apathy, depression	Apathy, depression
EMG	Mild axonal B/L lower limb motor neuropathy	Mild axonal B/L lower limb motor neuropathy
CK	56 U/L	720.0 U/L
LDH	210 U/L	180 U/L
ALT	9.0 IU/L	39 IU/L
AST	27.0 IU/L	63 IU/L
Acanthocyte ratio of CBC	Few not quantified	Few not quantified
MRI	Symmetrical atrophy of bilateral putamen and caudate nuclei with mildly increased T2W and FLAIR signal with subtle iron deposition	Symmetrical bilateral caudate atrophy
XK sequence changes	Not identified	Not identified
VPS13A sequence changes	c.799C > T	c.9263T > G
Chorein alterations	p.R267X	p.M3088R
Mutation types	Nonsense mutation (exon 11)	Missense mutation (exon 69)

+  +  + , severe; +  + , moderate; + , mild; *CK*, creatine kinase; *LDH*, lactate dehydrogenase; *ALT*, alanine transaminase/glutamic-pyruvic transaminase; *AST*, aspartate transaminase/glutamic–oxaloacetic transaminase; *EMG*, electromyography; *CBC*, complete blood count; *X*, termination codon

#### Case 2

The second case is a 28-year-old male born to non-consanguineous, healthy parents with no family history of illness. The patient had a history of multiple episodes of nocturnal generalized tonic clonic seizures for the last 6 years. Upon starting sodium valproate, the patient remained asymptomatic for seizures; however, presented an insidious onset of progressive dyskinetic movements of oro-lingual region for the last 3 years. On examination, the patient showed predominant and continuous involuntary movement of the jaw, tongue with swallowing difficulty. Choreiform movements were observed in the face, perioral region, and tongue. Lingual dysarthria and perioral dyskinesia with continuous pouting, chewing, protrusion, and jaw clenching movements were observed. Occasional choreiform movements in the limbs were also observed. In addition, the patient presented hyporeflexia in bilateral upper and lower limbs. EMG showed mild axonal motor neuropathy in bilateral lower limb. Frontal lobe dysfunction with symptoms of apathy and depression was also observed. There were no pyramidal or ataxic findings. Laboratory investigations showed an ALT level in the normal range (39 IU/L), while AST (63 IU/L) and CPK (720.0 U/L) levels were high. Lactate dehydrogenase level was normal (180 U/L) (Table 1). MRI brain report showed bilateral symmetrical bilateral caudate atrophy (Fig. 1A). Peripheral blood smear test showed the presence of acanthocytes (Fig. 1B). WES did not reveal mutations in the *XK* gene but a novel homozygous mutation (c.9263T > G; p.M3088R) in the *VPS13A* gene, confirming the patient as a case of ChAc.

### Genetic studies

WES followed by variant prioritization, in silico analysis for pathogenicity, and confirmation by Sanger sequencing revealed a previously reported (http://ncbi.nlm.nih.gov/clinvar/variation/373362) homozygous stop-gain mutation on the 11th exon of *VPS13A* gene in case 1, and a novel missense mutation on the 69th exon of *VPS13A* gene in case 2 (Table 2; Fig. 1C). The stop-gain mutation (c.799C > T; p.R267X) identified in case 1 affects all four major isoforms of *VPS13A* and results in premature termination of the protein (Fig. 1D). The ACMG guidelines classify this variant as pathogenic (PVS1 + PS1 + PM2 + PM2 + PP3 + PP4 + PP5) (Richards et al. 2015). Although the variant is a known pathogenic mutation, it was not previously associated with ChAc patients. The novel homozygous mutation (c.9263T > G; p.M3088R) identified in case 2 affects the conserved residue at the C-terminus of VPS13A (Fig. 1E) and is predicted as a pathogenic variant by various in silico prediction tools. The variant is likely pathogenic (PM2 + PM6 + PP3 + PP4) according to ACMG guidelines (Richards et al. 2015). This variant was identified only in two isoforms of *VPS13A*; however, the affected isoforms are highly expressed in all tissues, including different compartments of the brain (Online Resource 2).

**Table 2:** Details of the mutations in *VPS13A* gene identified in case 1 and case 2 and output of in silico pathogenicity prediction

	Case 1	Case 2
Position	chr9:79834914	chr9:80018225
Ref	C	T
Alt	T	G
Genotype	T/T	G/G
Gene.refgene	*VPS13A*	*VPS13A*
Func.refgene	Exonic (exon 11)	Exonic (exon 69)
ExonicFunc.refgene	Stop gain	Nonsynonymous SNV
AAChange.refgene	NM_001018037:exon11:c.799C > T: p.R267X NM_001018038:exon11:c.799C > T: p.R267X NM_015186:exon11:c.799C > T: p.R267X NM_033305:exon11:c.799C > T: p.R267X	NM_001018037:exon68:c.9146T > G:p.M3049R NM_033305:exon69:c.9263T > G: p.M3088R
avsnp150 status	Known (rs771004767)	Novel
SIFT_pred	⋅	D (0.001)
Polyphen2_HDIV_pred	⋅	D (0.997)
Polyphen2_HVAR_pred	⋅	D (0.921)
LRT_pred	D (0)	D (0)
MutationTaster_pred	A (1)	D (1)
MutationAssessor_pred	⋅	M (2.445)
FATHMM_pred	⋅	T (0.77)
PROVEAN_pred	⋅	D (−3.82)
VEST3_score	⋅	0.889
MetaSVM_pred	⋅	T (−0.185)
MetaLR_pred	⋅	T (0.443)
M-CAP_pred	⋅	D (0.117)
CADD_phred	36	32
DANN_score	0.997	0.987
fathmm-MKL_coding_pred	D (0.867)	D (0.974)
GERP + + _RS	1.4	5.77
phyloP100way_vertebrate	2.375	6.781
phyloP20way_mammalian	0.935	1.061
phastCons100way_vertebrate	0.991	1
SiPhy_29way_logOdds	11.503	15.756
SiPhy_29way_logOdds_rankscore	0.495	0.777
CLINSIG	Pathogenic	⋅
ACMG classification	Pathogenic (PVS1 + PS1 + PM2 + PM2 + PP3 + PP4 + PP5)	Likely pathogenic (PM2 + PM6 + PP3 + PP4)

*D* damaging; *T* tolerated; *A* known to be deleterious; *M* medium effect

Neurodegenerative conditions are multigene disorders that manifest diverse phenotypes among the affected individuals (Roberts et al. 2020). These can be attributed to mutations that occur in several functionally overlapping genes in similar or distinct pathways (Mitchell et al. 2021; Park and Neiman 2020). VPS13A is classified as a membrane trafficking protein (De et al. 2017; Kumar et al. 2018); hence, membrane trafficking proteins in the exome data of the cases were identified and variants in those genes were shortlisted irrespective of being in a homozygous or heterozygous state (Online Resource 3). Additionally, the proteins interacting with VPS13A were identified (Online Resource 4), and mutations in those genes were shortlisted if present in the exome data of the cases. Case 1 showed a heterozygous variant in the gene encoding VAMP-associated protein A (*VAPA*), albeit the variant (c.421A > G; p.Ile141Val) was predicted to be benign (BP4 + BP6) according to ACMG guidelines (Richards et al. 2015) (Online Resource 3; Online Resource 5).

Approximately, 54% of *VPS13A* is constituted by interspersed repeat sequences (Tomiyasu et al. 2012), thus rendering the gene highly susceptible to double stand breaks and rearrangements as well as mutations because of imperfect repair by non-homologous end joining. Insertions and heterozygous deletions have been reported in subjects with ChAc (Tomiyasu et al. 2012). Hence, whole exome data of cases were analyzed for CNVs and further annotated (Online Resource 6). To eliminate the false positive bias caused by variation in amplification during library preparation, the cut-off was set at copy number > 3 and loss of both alleles. The CNVs predicted as pathogenic or likely pathogenic were further assessed. We did not find any significant copy number variants in both cases (Online Resource 7).

Mitochondrial dysfunction is reported in ChAc; however, mutations in its mtDNA remain to be established (Kumar et al. 2018; Park et al. 2016; Yeshaw et al. 2019). To understand the mutation spectrum of mtDNA that may further exaggerate the phenotype during ChAc, we performed mitochondrial genome sequencing for both cases (Online Resource 6). Mitochondrial genome sequencing revealed that case 1 and case 2 belong to R30a1c and U2c haplogroups respectively. We observed a novel mutation in the *RNR2* gene encoding 16S rRNA (m.3144A > G) in case 1, while case 2 showed a novel mutation in *RNR2* gene (m.3208C > T) as well as a previously described DEAF phenotype-associated variant in *RNR1* gene that encodes 12S rRNA (m.742T > C). Besides, case 2 showed novel variants in protein coding genes, ATP synthase membrane subunit 6 (*ATP6*) (m.9109A > G; p.I195V) and cytochrome b (*CYB*) (m.15825C > T; p.T360M) but they were predicted to be benign. However, both the cases showed one common haplogroup-specific variant of the NADH dehydrogenase 2 (*ND2*) gene, m.4769A > G which has been previously associated with mitochondrial myopathy (Online Resource 8).

**Fig. 2:**
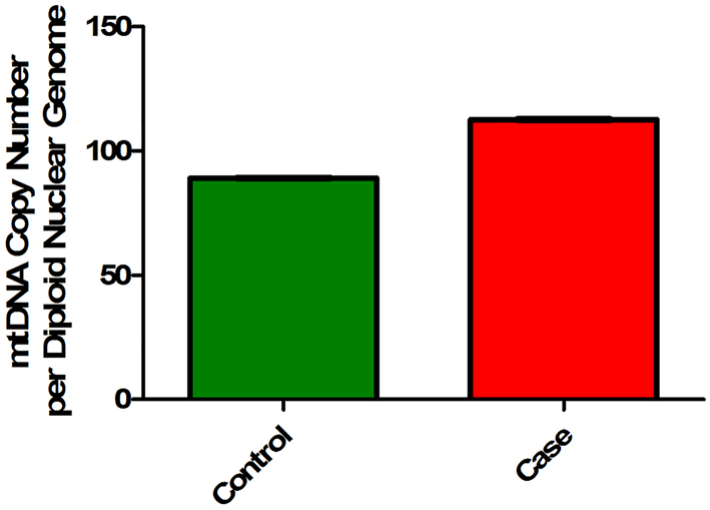
Relative mtDNA copy number in case 2. Bar diagram showing the mtDNA copy number relative to nuclear DNA copy number. The relative mtDNA copy number in case 2 was estimated to be 1.2-fold higher than that of the control subject

TaqMan chemistry-based quantitative real-time PCR was utilized to determine the effect of the novel *VPS13A* mutation p.M3088R on the mtDNA copy number in case 2 and control. The mtDNA copy number relative to the nuclear DNA copy number was computed in both case 2 and control subjects. The relative mtDNA copy number in case 2 was estimated to be 1.2-fold higher than that of the control subject (Fig. 2).

### In silico protein studies

**Fig. 3:**
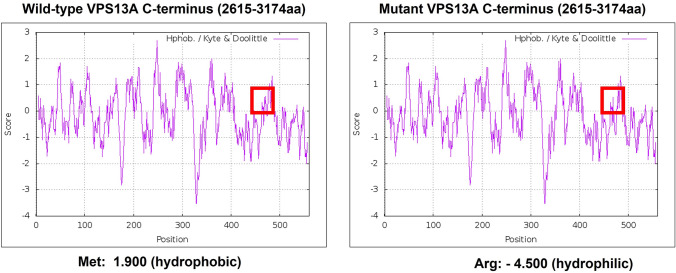
Hydrophobicity profile of C-terminus (2615–3174aa) region of VPS13A. The substitution of hydrophobic methionine for hydrophilic arginine at the 474th position (corresponding to the 3088th position of the full length VPS13A protein) alters the hydrophobicity profile of the VPS13A

The stop-gain mutation observed in case 1 results in an aberrant VPS13A protein with 267 amino acid residues instead of 3174 residues (Fig. 1D). Effect of missense mutation, p.M3088R, identified at the C-terminus region that is important for mitochondrial localization needs further study. The hydrophobicity profiling suggests a slight alteration in the protein folding because of the substitution of wildtype hydrophobic methionine with hydrophilic arginine residue at the 3088th position [corresponding to the 474th position in the C-terminus (2615–3174aa)] (Fig. 3). Since the structure for human VPS13A is not yet determined, we performed ab initio structure prediction with the trRossetta server using wildtype C-terminus protein sequence of hVPS13A protein. Further, the ab initio structure of wildtype C-terminus of the VPS13A was used as a template for homology modeling of the same region of human C-terminus of the VPS13A protein with the p.M3088R [~ p.M474R] mutation. Both wildtype and mutant model structures of the VPS13A C-terminus were assessed with the Ramachandran plot. Approximately 95.2% and 96.77% of the residues were in Ramachandran favored regions in wildtype and mutant structures, respectively (Online Resource 9). Structural alignment of wildtype and mutant models showed no major difference in structure conformation with a root mean square deviation (RMSD) of 0.463 (Online Resource 10).

**Fig. 4:**
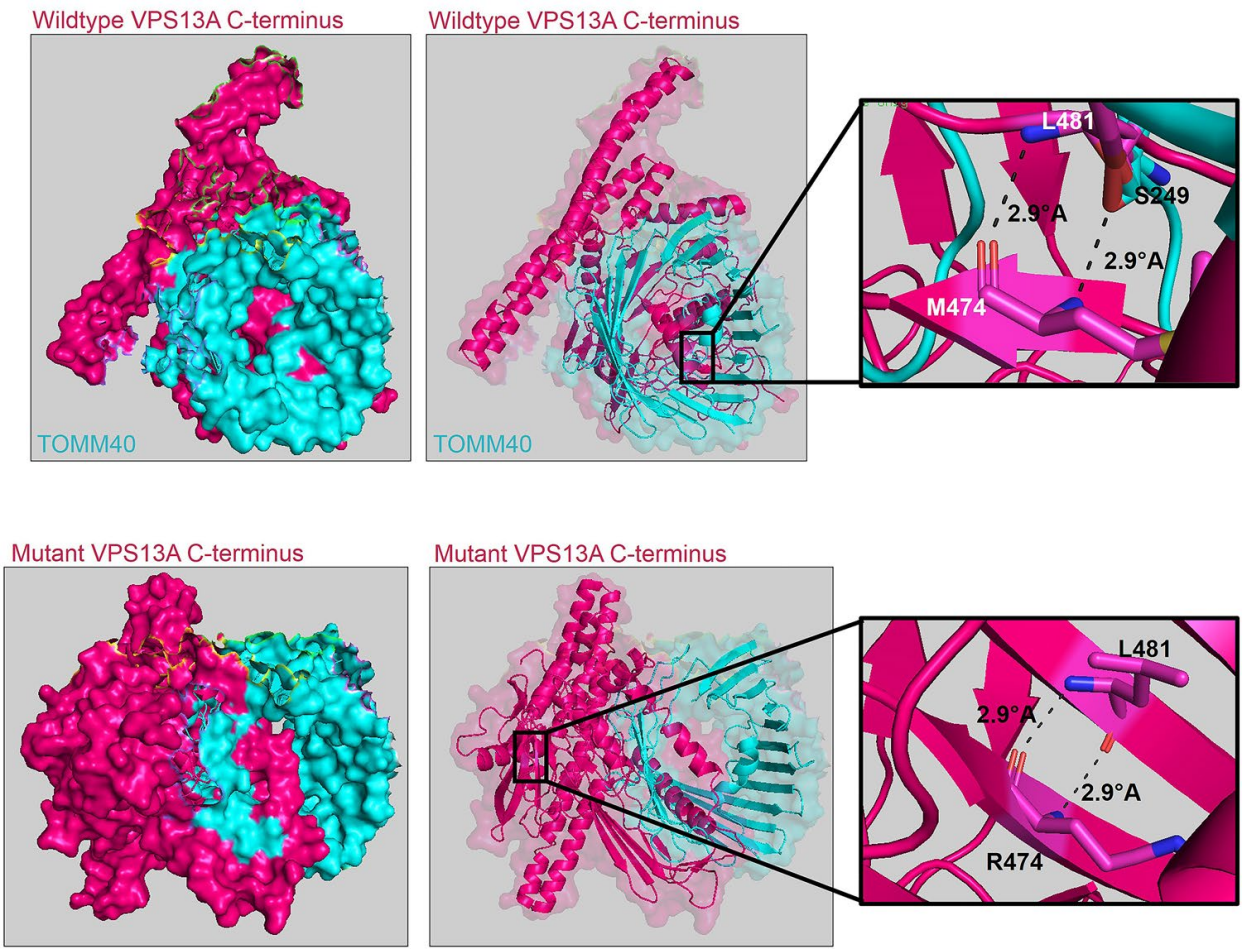
Molecular docking of wildtype and mutant VPS13A C-terminus with TOMM40. The p.M474 residue in wildtype VPS13A C-terminus forms an intramolecular polar bond with p.L481 and an intermolecular polar bond with p.S249 of TOMM40, whereas the mutant p.R474 residue fails to interact with TOMM40

VPS13A is reported to interact with TOMM20 and TOMM22 (Liu et al. 2018). To evaluate the effect of the p.M3088R (p.M474R) mutation that resides within the Pleckstrin homology (PH domain) of the VPS13A C-terminal region, we performed molecular docking with the PatchDock tool using modeled wildtype and mutant VPS13A C-termini with full length TOMM20 and TOMM22 protein structures. We did not observe a direct interaction of p.M3088 or p.M3088R with TOMM20 and TOMM22. We also performed a molecular docking using the TOM complex (PDB ID: 7CK6) as the receptor and our modeled wildtype or mutant VPS13A C-terminal as the ligand. Our prediction analysis revealed that the methionine at the 474th position of wildtype C-terminus of the modeled VPS13A forms a polar bond with the serine at the 249th position of TOMM40, while no bond formation was observed between the mutant arginine at the 474th position and TOMM40 (Fig. 4). However, we have conducted only functional predictions and these observations need to be experimentally validated.

## Discussion

The importance of ubiquitously expressed VPS13A protein in the organism is evident by the impact of mutations on major hematological and neurological functional loss leading to choreoacanthocytosis (Velayos Baeza et al. 2019; Kurano et al. 2007; Lang et al. 2017). Our study in two unrelated individuals identified two disparate mutations in *VPS13A*; one in the N-terminus (c.799C > T; p.R267X) that causes homozygous pathogenic nonsense mutation and the other at the C-terminus (c.9263T > G; p.M3088R) that disrupts interaction with the mitochondria. In addition, WES also identified key mutations in the interacting partners of VPS13A and, thus, may contribute to the wide spectrum of clinical phenotypes observed in the ChAc patients.

VPS13A is a member of the evolutionarily conserved gene family, Vps13, and is involved in diverse cellular functions including trafficking, vesicular transport and fusion, mitochondrial homeostasis, sporulation, phagocytosis, dopamine release, granule secretion and aggregation of blood platelets, calcium homeostasis, autophagy, cytoskeleton organization, phosphoinositide regulation, cell survival, and more (Velayos Baeza et al. 2019; Lang et al. 2017). Activity of VPS13A is influenced by two signaling pathways: the Lyn kinase pathway and the PI3K signaling pathway, and perturbation of either correlates with phenotypes of ChAc (Lang et al. 2017). The Lyn kinase pathway regulates autophagy, cytoskeleton dynamics, and synaptic plasticity, while PI3K and its downstream signaling proteins regulate diverse cellular activities, including regulation of actin polymerization and anti-apoptotic response (Lang et al. 2017). Most of the cases of ChAc, including our case 2, show seizures as one of the clinical phenotypes. The downstream proteins of the PI3K signaling pathway, activate serum and glucocorticoid inducible kinase (SGK1), which is known to target the Na^+^/K^+^-ATPase pump. The Na^+^/K^+^-ATPase pump modifies the potential of the cell membrane by electrogenic transport and enhancing the K^+^ conductance of the cell membrane. SGK1 is significantly reduced in cortical neurons differentiated from iPSCs generated from fibroblasts of ChAc patient. Thus, decrease in Na^+^/K^+^ pump capacity decreases cell membrane potential in ChAc neurons, thereby fostering excitation that triggers epileptic seizures in some ChAc patients (Hosseinzadeh et al. 2020).

VPS13A is recently recognized as a lipid transport protein (Kumar et al. 2018; Yeshaw et al. 2019; Dziurdzik and Conibear 2021). It is a peripheral membrane protein that localized to endoplasmic reticulum (ER)–mitochondria, ER–lipid droplets (LD), and mitochondria–endosome contact sites in human cells (Yeshaw et al. 2019). VPS13A consists of multiple domains that contribute toward this function (Dziurdzik and Conibear 2021). The N-terminal region, along with the SHR-binding domain (SHR_BD) and aberrant pollen transmission 1 (APT1) domain, interacts with lipids, including phosphoinositides (PIPs), and guides them to target organelles for further recruitment of other proteins (Dziurdzik and Conibear 2021). A large loop adjacent to the N-terminal consists of FFAT (two phenylalanines in an acidic tract) motif that interacts with the VAPA of the ER membrane (Kumar et al. 2018; Murphy and Levine 2016). The Vps13 adaptor binding (VAB) domain of VPS13A is important for its localization to different organelles via an adaptor protein (Kumar et al. 2018; Dziurdzik and Conibear 2021). The C-terminal region of VPS13A includes the ATG_C domain, domain reminiscent of the DH domain (DH-like domain; DH-L), and PH domain, which are important for mitochondrial and LD localization (Kumar et al. 2018; Yeshaw et al. 2019; Dziurdzik and Conibear 2021). Considering the importance of each domain of VPS13A in association with various molecules and their involvement in diverse cellular functions, the stop-gain mutation (c.799C > T; p.R267X) that is located at the N-terminus of VPS13A results in loss of function because of the truncated protein, leading to the ChAc phenotype.

Even though the alteration of conserved residue as well as in silico analysis suggests the deleterious effect of the novel mutation identified at the C-terminus, the functional effect is uncertain. The novel mutation (c.9263T > G; p.M3088R) was identified in a case that showed a history of multiple episodes of seizure. Recently, Luo et al. (2021) reported a homozygous nonsense variant (c.8282C > G; p.S2761X) at the C-terminus of the VPS13A gene in a ChAc patient with an epilepsy phenotype (Luo et al. 2021). Whether the variant at C-terminus of VPS13A is responsible for the epilepsy phenotype remains to be delineated. The C-terminal region of VPS13A (2615–3174aa) is demonstrated to be important for mitochondrial localization (Kumar et al. 2018; Dziurdzik and Conibear 2021). The medium spiny neuron (MSN) of ChAc displays shortened mitochondrial length, a reduced number of mitochondria, and mitochondrial hyperpolarization. Other homeostasis functions of mitochondria such as fission, fusion, and mitophagy were also affected (Yeshaw et al. 2019; Glaß et al. 2018). The C-terminal region of VPS13A consists of the ATG_C domain, the DH-L domain, and the PH domain (Kumar et al. 2018). PH domain along with ATG-C domain is reported to localize to mitochondria. Similarly, the PH domain of p210 BCR-ABL is shown to bind to mitochondrial phospholipid, cardiolipin, and mediate its localization to mitochondria (Shimasaki et al. 2018). Although the DH-L and PH domains have mitochondrial binding and lipid binding regions (Kumar et al. 2018), the mechanism by which VPS13A localized to mitochondria is unclear. A recent study by Liu et al. (2018) has identified interactions of the VPS13A with mitochondrial outer membrane proteins TOMM20 and TOMM22. However, we did not observe direct interaction of wildtype or mutant residues of VPS13A with TOMM20 and TOMM22 molecular docking. TOMM22 is part of the pre-protein translocase complex (TOM complex), located on the outer membrane of mitochondria, and this complex includes other proteins such as TOMM5, TOMM6, TOMM7, TOMM40, and TOMM70 (Wang et al. 2020). Docking of modeled wildtype and mutant VPS13A C-termini with the TOM core complex showed interactions of wildtype VPS13A C-termini with the TOMM40 mediated by bond formation between p.M3088 of VPS13A and S249 of TOMM40. ATG2A proteins containing the ATG_C domain are known to localize to the ER–mitochondrial contact site and recruitment to the contact site involves binding to TOMM40 (Tang et al. 2019). Disruption of this interaction in the p.M3088R mutant suggests a potential inability to localize to the mitochondrial outer membrane, which might perturb lipid transfer from the mitochondria to the ER and LD.

Mitochondrial homeostasis is maintained by (a) mitochondrial dynamics (fusion and fission) and (b) mitophagy. Dysfunctional mitochondria are segregated and depolarized during its fission event. Depolarized mitochondria are a prerequisite for mitophagy which further eliminates dysfunctional mitochondria (Twig and Shirihai 2011; Rong et al. 2021). VPS13A-depleted cells show decreased fusion, increased fission, and mitochondrial depolarization but impaired mitophagy (Park et al. 2016; Yeshaw et al. 2019), which might partially explain our observation of increased mtDNA copy number in case 2 with the C-terminal exon 69 mutation. Mitochondrial dysfunction in the absence of VPS13A increases stress and ROS production, which, in turn, causes the transfer of lipids from neuron to glial cells where LDs are formed (Liu et al. 2017). VPS13A depleted cells reported an increase in the number of LDs which could be due to disruption of turnover of LDs as ER cannot associate with LDs in the absence of VPS13A (Yeshaw et al. 2019). Thus, the formation and accumulation of LDs without turnover result in an overall imbalance in lipid homeostasis and other metabolic pathways that progressively affect the cellular function of glial and neuronal cells, resulting in neurodegeneration (Yeshaw et al. 2019).

McLeod syndrome, caused by mutations in *XK* gene, show an overlapping phenotype with ChAc (Peikert et al. 2022). We ruled out the possibility that our cases were McLeod syndrome, as our exome sequencing did not identify any mutation in the *XK* gene. Recently, co-localization of VPS13A with XK and their indirect interaction via an unknown protein to form a VPS13A-XK complex were reported (Park and Neiman 2020). Thus, mutations in VPS13A interacting proteins or proteins belonging to a similar class and participating in the same molecular pathway may promote the ChAc phenotype either independently or through co-mutation. Our exome data analysis revealed a heterozygous benign mutation in *VAPA* along with a homozygous pathogenic mutation in *VPS13A* in case 1. VAPA is an ER membrane protein that is known to interact with the FFAT motif of VPS13A to tether to ER (Kumar et al. 2018); thus, it is likely that co-mutation in both the interacting genes may exacerbate the disease phenotype. Diverse phenotypes observed among the neurodegenerative disorders such as ChAc is more likely because of the co-mutations in multiple genes (Roberts et al. 2020). Other homozygous variants identified by exome sequencing in both cases (Online Resource 3) may directly or indirectly associate with additional phenotypes displayed in ChAc patients. A concerted study is warranted to elucidate mechanisms and functions of identified variants.

In summary, two cases of ChAc with significant clinical heterogeneity were identified. The c.799C > T; p.R267X homozygous variant in *VPS13A* was identified in case 1 with Parkinsonism as an additional phenotype. In our second case with seizure as an additional phenotype, a homozygous missense variant (c.9263T > G; p.M3088R) in *VPS13A* was identified. This is the first report of the variant (c.9263T > G) in a ChAc patient. Although we could not study the expression levels of chorein at the protein level, extensive molecular and bioinformatics analysis strongly supports the deleterious impact of the identified novel mutation. In silico analysis with p.M3088R showed a loss of interaction between VPS13A and TOMM40. Additionally, we observed an increase in mitochondrial DNA copy number in case 2, which could be because of decreased mitophagy commonly observed in VPS13A depleted cells. Hence, p.M3088R may be associated with the deregulated mitochondrial homeostasis; however, the lack of a cell model constrained our study, and further functional analysis to support the observation is warranted.

## Supplementary Information

Below is the link to the electronic supplementary material.

Supplementary file 1 Details of primer designed for validation of variants by Sanger Sequencing (DOCX 20 KB)

Supplementary file 2 Expression of isoforms of *VPS13A* in human tissues. Four major isoforms of *VPS13A* (A) and their expression represented as transcripts per million (TPM) in different tissues of human origin. The transcript ENST00000360280.7 (NM_033305) encodes for *VPS13A*, which is ubiquitously expressed in all tissues with the highest expression levels in the brain (TIF 122759 KB)

Supplementary file 3 Information of shortlisted candidate genes and variants identified in whole exome sequencing for both presented cases of choreoacanthocytosis (XLSX 629 KB)

Supplementary file 4 VPS13A interacting protein. Protein-Protein interaction network for VPS13A, retrieved from the Human Integrated Protein Protein Interaction rEference (HIPPIE) database (TIF 38183 KB)

Supplementary file 5 Validation of *VAPA* variant. Sanger sequencing confirms the presence of a heterozygous variant (c.421A>G; p.Ile141Val) in the *VAPA* gene in case 1 (TIF 10880 KB)

Supplementary file 6 Methods employed for copy number variation analysis and mitochondrial genome sequencing (DOCX 15 KB)

Supplementary file 7 Copy number variant information obtained from EXCAVATOR were further annotated by ClassifyCNV (XLSX 19 KB)

Supplementary file 8 Information on shortlisted candidate genes and variants identified in mitochondrial genome sequencing in both cases of choreoacanthocytosis (XLSX 15 KB)

Supplementary file 9 Ramachandran plot. Structure assessment of the modeled wildtype and mutant VPS13A C-terminus shows that about 95.2% and 96.77% of the residues are in Ramachandran favored regions in wild type and mutant proteins, respectively (TIF 106569 KB)

Supplementary file 10 Three-dimensional superimposition of wildtype and mutant modeled structures of VPS13A C-terminus (2615-3174aa). A superimposed modeled structure of the wildtype C-terminus of VPS13A containing wildtype methionine at the 474th position (corresponding to the 3088th position of full length VPS13A) and the mutant C-terminus of VPS13A containing arginine at the 474th position, did not show major alterations in the conformation of the protein (TIF 99730 KB)

## Data Availability

The datasets generated during and/or analyzed during the current study are available from the corresponding author on reasonable request. The variants identified in the study have been submitted to ClinVar database (https://www.ncbi.nlm.nih.gov/clinvar/). The accession numbers SCV002526445 and SCV002526446 correspond to variants c.799C > T (p.R267X) and c.9263T > G (p.M3088R), respectively.
