# Supplementary material for: Exome sequencing of choreoacanthocytosis reveals novel mutations in VPS13A and co-mutation in modifier gene(s)
Source: Mol Genet Genomics. 2023 May 20;298(4):965–76. doi: 10.1007/s00438-023-02032-2 (PMC10227119; doi:10.1007/s00438-023-02032-2)
Supplement: Supplementary file 1 — Details of primer designed for validation of variants by Sanger Sequencing (DOCX 20 KB) [file 438_2023_2032_MOESM1_ESM.docx]

**Online Resource 1 (ESM_1):** Details of primer designed for validation of variants by Sanger Sequencing

| **Mutation location** | **Primer** | **Primer sequence** | **Tm** | **Amplicon size** | **Amplicon region** |
| --- | --- | --- | --- | --- | --- |
| chr9:79834914 | VPS13AF | TGTGAACATCTGGGAACTGT | 60°C | 538bp | chr9:79834741-79835278 |
|  | VPS13AR | AGAGGCACATCAGGTTTGAA |  |  |  |
| chr9:80018225 | VPS13AF2 | TTAATGGTTTGCCCCATTGAGA | 60°C | 403bp | chr9:80018041-80018443 |
|  | VPS13AR2 | TCTGTGCATGACTGTGTTTATCTG |  |  |  |
| chr18:9944924 | VAPAF | CAGAAACTACTTTTGCTTTAAC | 60°C | 403bp | chr9:80018041-80018443 |
|  | VAPAR | CTCATTGAAGTGACAGTCGGAGC | 64°C | 223bp | chr18:9944742-9944964 |
