# Supplementary figures and images for: Exome sequencing of choreoacanthocytosis reveals novel mutations in VPS13A and co-mutation in modifier gene(s)
Source: Mol Genet Genomics. 2023 May 20;298(4):965–76. doi: 10.1007/s00438-023-02032-2 (PMC10227119; doi:10.1007/s00438-023-02032-2)

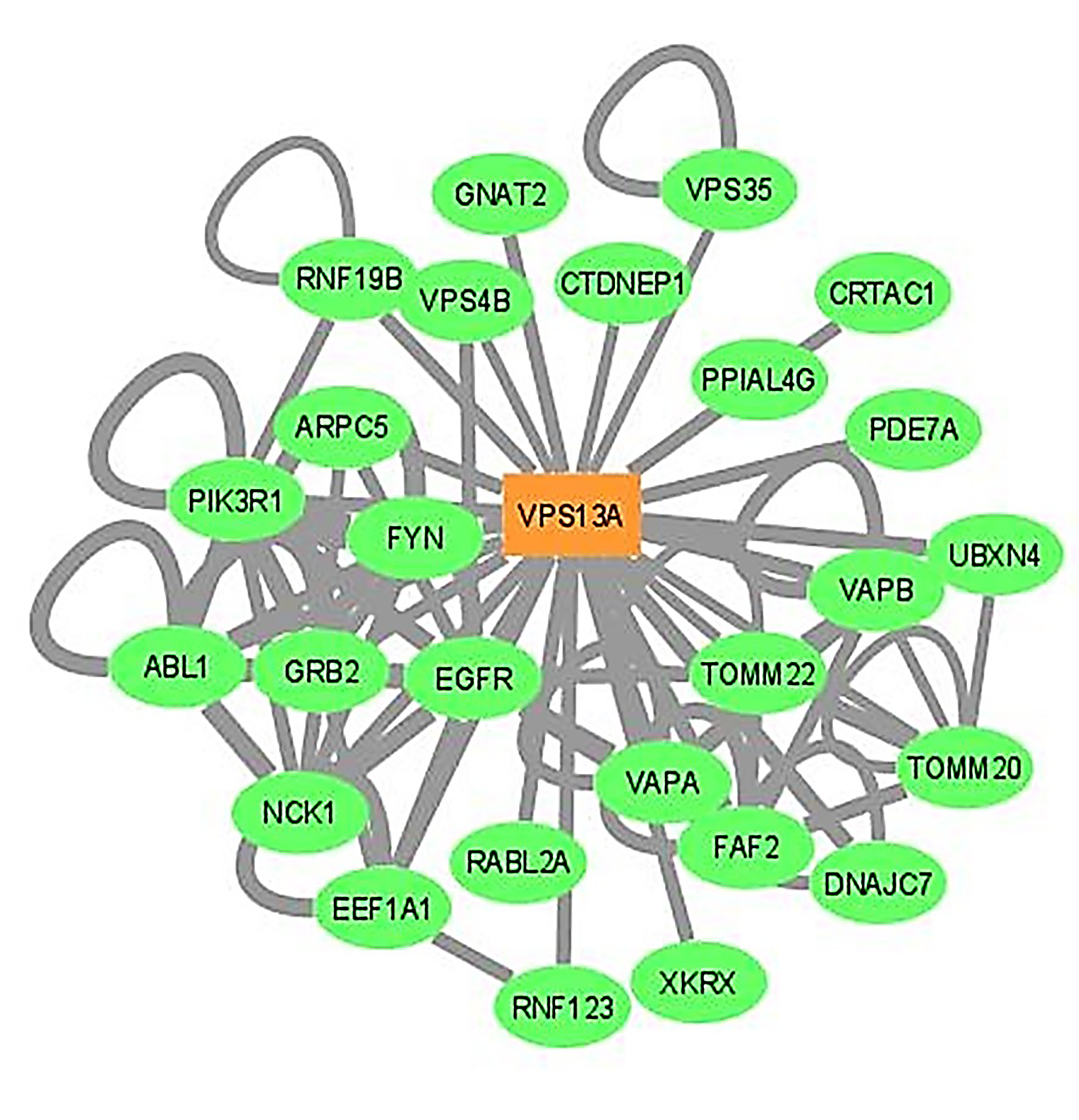

Supplement: Supplementary file 4 — VPS13A interacting protein. Protein-Protein interaction network for VPS13A, retrieved from the Human Integrated Protein Protein Interaction rEference (HIPPIE) database (TIF 38183 KB) [file 438_2023_2032_MOESM4_ESM.tif]

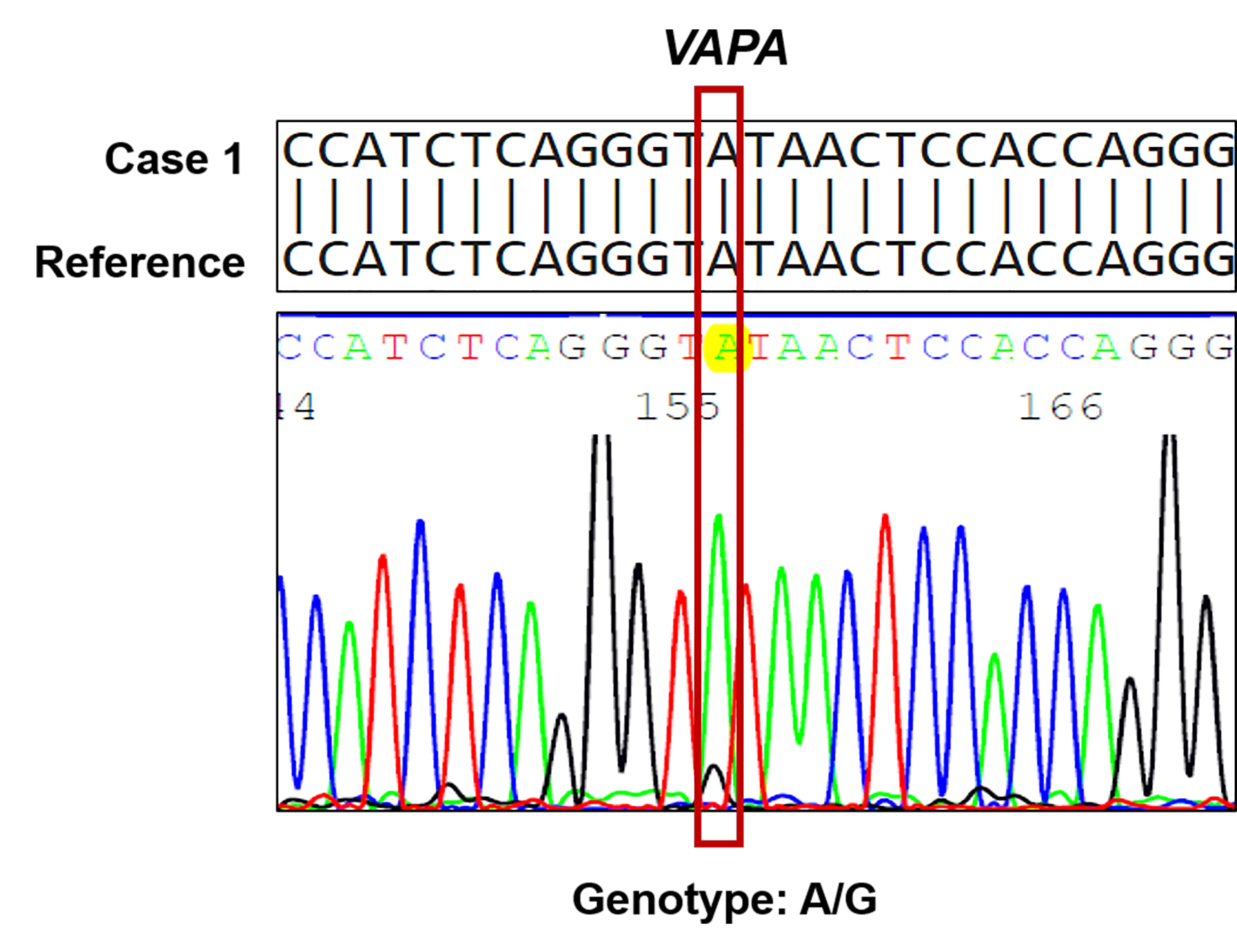

Supplement: Supplementary file 5 — Validation of VAPA variant. Sanger sequencing confirms the presence of a heterozygous variant (c.421A>G; p.Ile141Val) in the VAPA gene in case 1 (TIF 10880 KB) [file 438_2023_2032_MOESM5_ESM.tif]
