# Supplementary material for: Exome sequencing of choreoacanthocytosis reveals novel mutations in VPS13A and co-mutation in modifier gene(s)
Source: Mol Genet Genomics. 2023 May 20;298(4):965–76. doi: 10.1007/s00438-023-02032-2 (PMC10227119; doi:10.1007/s00438-023-02032-2)
Supplement: Supplementary file 6 — Methods employed for copy number variation analysis and mitochondrial genome sequencing (DOCX 15 KB) [file 438_2023_2032_MOESM6_ESM.docx]

**Copy number variation (CNV) analysis**

EXCAVATOR2 was employed to identify the copy number variants from WES data obtained from the Ion torrent platform (D'Aurizio et al. 2016). Further, the FastCallResult file obtained from EXCAVATOR2 was annotated and classified using ClassifyCNV (Gurbich and Ilinsky 2020).

**Mitochondrial genome sequencing and data analysis**

The library preparation and sequencing of the whole mitochondrial genome were performed as previously described by Mani et al. (2019) on the Ion Torrent Personal Genome Machine (PGM) System (ThermoFisher Scientific, USA). Mtool box was utilized for alignment, haplogroup prediction, and annotation of sequenced data (Calabrese et al. 2014). The variants were further segregated as “Haplogroup specific” variants and “Private” variants. The haplogroup specific variants previously associated with disease were shortlisted for further analysis. The non-synonymous private variants were referred to MitoMap (www.mitomap.org) and MitImpact databases (www.mitimpact.css-mendel.it/) to identify their status. Also, the variants with a heteroplasmy fraction >0.01, and nucleotide variability of <0.0026 or a disease score of >0.4311 were shortlisted for further analysis (Mani et al. 2019).

References:

1. D'Aurizio R, Pippucci T, Tattini L, Giusti B, Pellegrini M, Magi A (2016) Enhanced copy number variants detection from whole-exome sequencing data using EXCAVATOR2. Nucleic Acids Res 44:e154. <https://doi.org/10.1093/nar/gkw695>
2. Gurbich TA, Ilinsky VV (2020) ClassifyCNV: a tool for clinical annotation of copy-number variants. Sci Rep 10:20375. https://doi.org/10.1038/s41598-020-76425-3
3. Calabrese C, Simone D, Diroma MA, Santorsola M, Guttà C, Gasparre G, et al (2014) MToolBox: a highly automated pipeline for heteroplasmy annotation and prioritization analysis of human mitochondrial variants in high-throughput sequencing. Bioinformatics 30:3115-7. https://doi.org/10.1093/bioinformatics/btu483
4. Mani MS, Chakrabarty S, Mallya SP, Kabekkodu SP, Jayaram P, Varghese VK, et al (2019) Whole mitochondria genome mutational spectrum in occupationally exposed lead subjects. Mitochondrion 48:60-66. https://doi.org/10.1016/j.mito.2019.04.009
